# Forms of Workplace Violence in Veterinary Organisations in Slovenia

**DOI:** 10.3390/healthcare14193357

**Published:** 2026-10-08

**Authors:** Miha Dvojmoč, Valentina Kubale, Ajda Šulc, Breda Jakovac Strajn, Ožbalt Podpečan

**Affiliations:** 1 Faculty of Criminal Justice and Security, University of Maribor, Kotnikova 8, 1000 Ljubljana, Slovenia; miha.dvojmoc@um.si (M.D.); ajda.sulc@um.si (A.Š.); 2 Veterinary Faculty, University of Ljubljana, Gerbičeva 60, 1000 Ljubljana, Slovenia; valentina.kubaledvojmoc@vf.uni-lj.si (V.K.); breda.jakovacstrajn@vf.uni-lj.si (B.J.S.)

**Keywords:** workplace violence, veterinary professionals, occupational violence, emotional responses, Slovenia

## Abstract

**Highlights:**

**What are the main findings?**

- To our knowledge, this is the first nationwide study to examine workplace violence involving animal owners, co-workers, and supervisors among employees of veterinary organisations in Slovenia.
- Workplace violence involving animal owners, co-workers, and supervisors was reported, with verbal and psychological forms particularly evident in interactions with animal owners.
- Fear, helplessness, and uncertainty were prominent emotional responses associated with reported workplace violence involving animal owners, while violence involving co-workers and supervisors was also associated with negative emotional responses.

**What are the implications of the main findings?**

- The results of this study provide important insight into the psychosocial risks of workplace violence faced by employees of veterinary practices and clinics in Slovenia and suggest that work experience, higher education level, and male gender are associated with less intense negative emotional responses.
- Given the lack of existing research in this area within the Slovenian context, these findings contribute to a better understanding of working conditions and challenges related to workplace violence in the veterinary profession.

**Abstract:**

Background/Objectives: Workplace violence is a recognised psychosocial risk across many professions but remains insufficiently investigated in veterinary practice. Evidence on workplace violence among veterinary employees in Slovenia is limited. This study aimed to examine reported exposure to different forms of workplace violence involving animal owners, co-workers, and supervisors among employees of veterinary organisations in Slovenia and to assess associated emotional responses. Methods: A cross-sectional online survey was conducted between December 2024 and April 2025 among employees of all 182 registered veterinary practices and clinics in Slovenia. Of 1351 eligible employees, 178 completed the questionnaire (overall response rate: 13.17%), including 155 of 873 eligible veterinarians (veterinarian response rate: 17.75%). Respondents rated their agreement with statements describing verbal, physical, and psychological violence involving animal owners, co-workers, and supervisors, as well as associated negative emotional responses. Data were analysed using one-sample and paired-samples *t*-tests, principal component analysis, and multiple linear regression. Results: Respondents reported exposure to workplace violence involving animal owners, co-workers, and supervisors, with verbal and psychological forms particularly evident in interactions with animal owners. Fear, helplessness, and uncertainty were prominent emotional responses associated with violence involving animal owners, while violence involving co-workers and supervisors was also associated with negative emotional responses. Greater work experience was significantly associated with less intense negative emotional responses across all three perpetrator groups. Higher education and male gender were additionally associated with less intense emotional responses to violence involving animal owners. Conclusions: Workplace violence represents an important psychosocial concern in Slovenian veterinary organisations and may originate from both external and internal sources. Training, written protocols, and organisational and psychological support may warrant consideration as potential preventive measures; however, their effectiveness was not evaluated in this study and requires further investigation. The low response rate and potential non-response and self-selection bias limit the generalisability of the findings. Further research using validated event-based measures and stronger recruitment strategies is warranted.

## 1. Introduction

Workplace violence is an important psychosocial occupational risk that may affect employee safety, well-being, and work performance. Although related concepts such as aggression, workplace violence, incivility, and bullying may overlap, they represent distinct forms of workplace behaviour. Aggression is commonly used as a broad term for behaviour intended to cause harm and may be verbal, psychological, or physical [1]. Workplace violence generally refers to more overt forms of threatening, intimidating, abusive, or physically harmful behaviour [2]. Workplace incivility is characterised by low-intensity disrespectful behaviour with ambiguous intent to harm [3], whereas workplace bullying involves repeated negative behaviour over time, typically accompanied by an actual or perceived power imbalance [4]. Distinguishing these constructs is important because they differ in severity, persistence, and underlying dynamics, although they may coexist or overlap within the same workplace.

Workplace violence and other forms of workplace mistreatment may originate both externally, from clients or service users, and internally, from co-workers, senior colleagues, or supervisors [2]. This distinction is particularly relevant in veterinary practice, where employees interact with animal owners who may be experiencing distress related to animal illness, injury, treatment costs, or difficult clinical decisions, while simultaneously working within demanding organisational and interpersonal environments [5,6,7,8]. Consequently, veterinary professionals may encounter verbal, psychological, and physical forms of inappropriate or violent behaviour from different sources.

Recent veterinary research increasingly demonstrates the importance of considering both the form and source of workplace mistreatment. Irwin and Hall (2022) [9] examined veterinarians’ experiences of client incivility, while Irwin et al. (2022) [10] extended this work by examining incivility originating from clients, co-workers, and senior colleagues. Subsequent studies showed that responses may differ according to the type and source of uncivil behaviour [11] and examined individual and organisational approaches to managing workplace incivility [12]. Other veterinary studies have investigated workplace bullying [13], occupational hazards and adverse workplace behaviours [14], threats and violence [6,7,15,16], and, more recently, aggression and violence originating specifically from animal owners as well as workplace harassment and transgressive behaviour within veterinary organisations [17,18]. Collectively, this literature indicates that workplace mistreatment in veterinary settings encompasses related but distinct behaviours and may arise from both external and internal sources.

Reported levels of workplace violence vary substantially across studies and countries. For example, studies from Finland, Germany, and Japan have reported differing levels of exposure to threatening or violent situations among veterinary professionals [6,7,15,16]. More recent nationwide research from Germany has also documented aggression and violence directed at veterinary professionals by animal owners [18]. However, estimates across these studies should not be directly compared because definitions of violence, study populations, measurement instruments, item wording, response formats, and recall periods differ. Rather than indicating simple geographical differences, this variability highlights the methodological complexity of assessing workplace violence in veterinary settings and the need for caution when comparing prevalence estimates across studies.

The consequences of workplace violence and other forms of workplace mistreatment may extend beyond immediate safety concerns. Previous veterinary research has reported associations between threatening situations or negative workplace behaviours and stress, fatigue, sleep disturbances, feelings of loneliness, and other adverse occupational and psychological outcomes [7,14,15]. Research on veterinary workplace incivility has similarly demonstrated that the source and nature of inappropriate behaviour may be relevant to employees’ perceptions, emotional responses, and coping strategies [9,10,11]. However, differences in terminology and measurement across studies mean that findings concerning incivility, bullying, aggression, and workplace violence should not be treated as directly equivalent.

Conceptually, emotional responses to workplace violence may vary according to both the source of the behaviour and characteristics of the individual experiencing it. The perpetrator’s relationship to the employee may influence how threatening, controllable, or personally significant an incident is perceived to be, while work experience, education, gender, and occupational role may be associated with differences in previous exposure, professional responsibilities, or resources for managing difficult interactions. These characteristics were therefore examined as potential correlates of emotional responses rather than as causal determinants. However, these relationships remain insufficiently understood in veterinary settings, and given the cross-sectional design of the present study, observed associations should not be interpreted as demonstrating causal relationships.

In Slovenia, workplace violence has previously been investigated in healthcare [19], policing [2,20], and public administration [21], whereas evidence concerning employees of veterinary practices and clinics remains limited. To our knowledge, no nationwide Slovenian study has simultaneously examined reported workplace violence involving animal owners, co-workers, and supervisors among employees of veterinary organisations together with the associated emotional responses. The present study therefore aimed to examine reported exposure to different forms of workplace violence involving these three perpetrator groups and to investigate the emotional responses associated with these experiences.

Specifically, the study addressed the following research questions: (a) What forms of workplace violence involving animal owners, co-workers, and supervisors are reported by employees of veterinary organisations in Slovenia? (b) What emotional responses are associated with reported workplace violence involving these perpetrator groups? (c) Are respondents’ demographic and occupational characteristics, including work experience, education, gender, and occupational role, associated with the intensity of reported emotional responses? (d) How do respondents perceive their knowledge, training, and organisational support for managing violent situations?

## 2. Materials and Methods

The study of workplace violence in veterinary clinics and practices in Slovenia aimed to gain insight into reported exposure to workplace violence, identify the forms of violence reported by employees, and examine associated emotional responses. A quantitative cross-sectional study was conducted using an online survey among employees of veterinary clinics and practices in Slovenia.

To gain insight into workplace violence in veterinary practices and clinics in Slovenia, a survey was conducted among employees. The questionnaire used in this study was adapted and modified for the veterinary context based on instruments and methodological approaches previously used in Slovenian research on occupational safety, workplace threats, aggression, and violence. Konkolič et al. (2016) [22] investigated perceived sources of occupational threats using a five-point Likert scale, including risks associated with workplace conditions and interpersonal relationships. Gabrovec and Lobnikar (2014) [23] examined workplace violence and organisational approaches to managing aggression and ensuring safety in healthcare settings, while Gabrovec et al. (2017) [24] used a structured questionnaire to assess different forms of workplace violence, associated emotional responses, knowledge of violence management, and training needs among healthcare personnel. For the present study, these methodological approaches were adapted to the veterinary setting, with terminology and relevant items modified to reflect interactions between veterinary employees and animal owners/clients, as well as workplace relationships involving co-workers and supervisors. Prior to data collection, the adapted questionnaire was reviewed and pre-tested by professional colleagues with relevant expertise to assess the comprehensibility, relevance, and appropriateness of the items and wording. Their feedback was used to refine the final version of the questionnaire. The adapted veterinary version was not subjected to a separate formal psychometric validation study prior to data collection.

Empirical data were collected using an online questionnaire in the 1ka web environment between 11 December 2024 and 1 April 2025. Respondents required approximately 10 min to complete the questionnaire. The collected data were then exported and analysed using IBM SPSS Statistics software, version 31. Within the descriptive statistics, frequencies and percentages for categorical variables and means and standard deviations for numerical variables are presented. All Likert-scale data (although ordinal) were treated as interval to compare the mean agreement scores, since the items used equally labelled response categories and the tests used are known to be robust to this treatment. To compare mean agreements of composite variables to the midpoint of 3 (“neither agree nor disagree”), one-sample *t*-tests were used, and paired-samples *t*-tests were used for comparison of means. Multiple linear regression was conducted to examine the associations between negative emotional responses and respondent characteristics, and Pearson correlation was calculated for numerical variables. Education was dichotomised into lower education (secondary school or bachelor’s degree) and higher education (MSc or PhD) and entered as a binary dummy variable, with lower education as the reference category. Gender and work group were also entered as dummy variables, with women and veterinarians, respectively, as the reference categories. Age was not included simultaneously with work experience because of the strong correlation between these variables and was therefore examined separately in unadjusted analyses. All tests were two-sided with α = 0.05.

### 2.1. Sample Description

Employees from all 182 veterinary practices and clinics registered in Slovenia were invited to participate in the study. At that time, a total of 873 veterinarians and 478 support staff were employed. Of 1351 eligible employees, 274 started the questionnaire and 178 completed it (overall response rate: 13.17%), including 155 of 873 eligible veterinarians (veterinarian response rate: 17.75%). The questionnaire did not collect information on respondents’ organisation names to protect their privacy, so an organisational-level response rate could not be calculated and clustering was not assessed. Among respondents, 30.5% were male and 69.5% female. The sample included respondents aged 21 to 78 years, with a mean age of 41.9 years (SD = 11.7), and work experience ranging from 0 to 40 years, with a mean of 16 years (SD = 11.4). Most held a bachelor’s degree (59.9%), with fewer holding an MSc degree (23.7%), a PhD (11.3%), and the fewest having only a secondary school qualification (5.1%). Most respondents were employed as veterinarians (87.1%), with 9% working as professional associates and 3.9% as teaching staff. Detailed characteristics of the sample are presented in Table 1.

### 2.2. Questionnaire and Variable Description

The questionnaire comprises three sets of closed-ended questions. Most required respondents to select an answer on a 5-point Likert scale, while some offered only “yes” or “no” response options. The questionnaire also included one open-ended question.

The first set addressed experiences of violence from three different actors: animal owners, co-workers, and supervisors. Respondents answered these questions on a 5-point agreement scale ranging from 1 (strongly disagree) to 5 (strongly agree), which was treated as interval data. Questions first asked the extent to which respondents agreed that they had experienced the listed forms of violence from animal owners in the past year, with three separate questions for verbal, physical, and psychological violence. For these variables, a one-sample *t*-test was conducted to determine whether the mean statistically significantly differed from the midpoint of 3. This was followed by a question concerning exposure to behaviours from co-workers, which did not distinguish by type of violence but instead asked about 29 different instances of violence in a single question. Respondents rated the extent to which they agreed that they had been exposed to each item, again on a scale from 1 (strongly disagree) to 5 (strongly agree). Similarly, respondents used the same scale to rate the same 29 instances of violence for their supervisors.

The second set included questions relating to the experience of specific emotions in a given situation. For each potential perpetrator of violence against respondents, participants were asked to rate the extent to which they experienced a particular emotion when confronted with violent behaviour from animal owners, co-workers, and supervisors. They used a scale from 1 (emotion not expressed at all) to 5 (emotion very strongly expressed), which we treated as an interval. All resulting variables are approximately normally distributed, with kurtosis and skewness between −2 and 2.

We conducted a principal components analysis (PCA) of the variables measuring negative emotional responses to violence involving animal owners, co-workers, and supervisors, with the aim of reducing the variables into a smaller number of components. The Kaiser–Meyer–Olkin (KMO) measure indicated adequate sampling adequacy (KMO = 0.908), and Bartlett’s test of sphericity was statistically significant (*p* < 0.001), supporting the suitability of the data for PCA. Four components had eigenvalues greater than 1; however, the fourth component had an eigenvalue of only 1.028 and was not substantively interpretable. Based on inspection of the scree plot and the interpretability of the solution, a three-component solution was retained. The third component had an eigenvalue of 1.323, and the three retained components together explained 68.3% of the total variance. Because the components were correlated, an oblique Direct Oblimin rotation was applied. Component loadings ≥0.40 are presented in Table 2. The three-component structure should be interpreted cautiously because the same seven emotional responses were assessed separately for each perpetrator group. Thus, the observed component structure may partly reflect the organisation of the questionnaire rather than distinct latent psychological constructs. PCA was therefore used primarily as an exploratory data-reduction procedure to support the calculation of summary emotional-response scores for each perpetrator group, rather than as a formal validation of latent constructs.

The first component presents variables measuring the emotions employees experience because of violence from superiors, the second from animal owners, and the third from co-workers. We created new composite variables by calculating the average scores for the relevant statements. Each new variable thus represents the average intensity of all listed negative emotions experienced in relation to a particular perpetrator, on a scale from 1 (the emotion is not expressed at all) to 5 (the emotion is very strongly expressed). The internal consistency of the resulting components is high: Cronbach’s alpha coefficient is 0.901 for animal owners, 0.938 for co-workers, and 0.960 for superiors.

The constructed components enabled us to compare how different groups of respondents experience the same negative emotions when subjected to violence from three different actors. In the third set, we asked respondents about their competence in dealing with violent customers or animal owners, using a scale from 1 (strongly disagree) to 5 (strongly agree), which we treated as a variable. The responses to all six statements are approximately normally distributed, with skewness and kurtosis values between −2 and 2.

## 3. Results

### 3.1. Experience of Individual Forms of Violence

**Figure 1:**
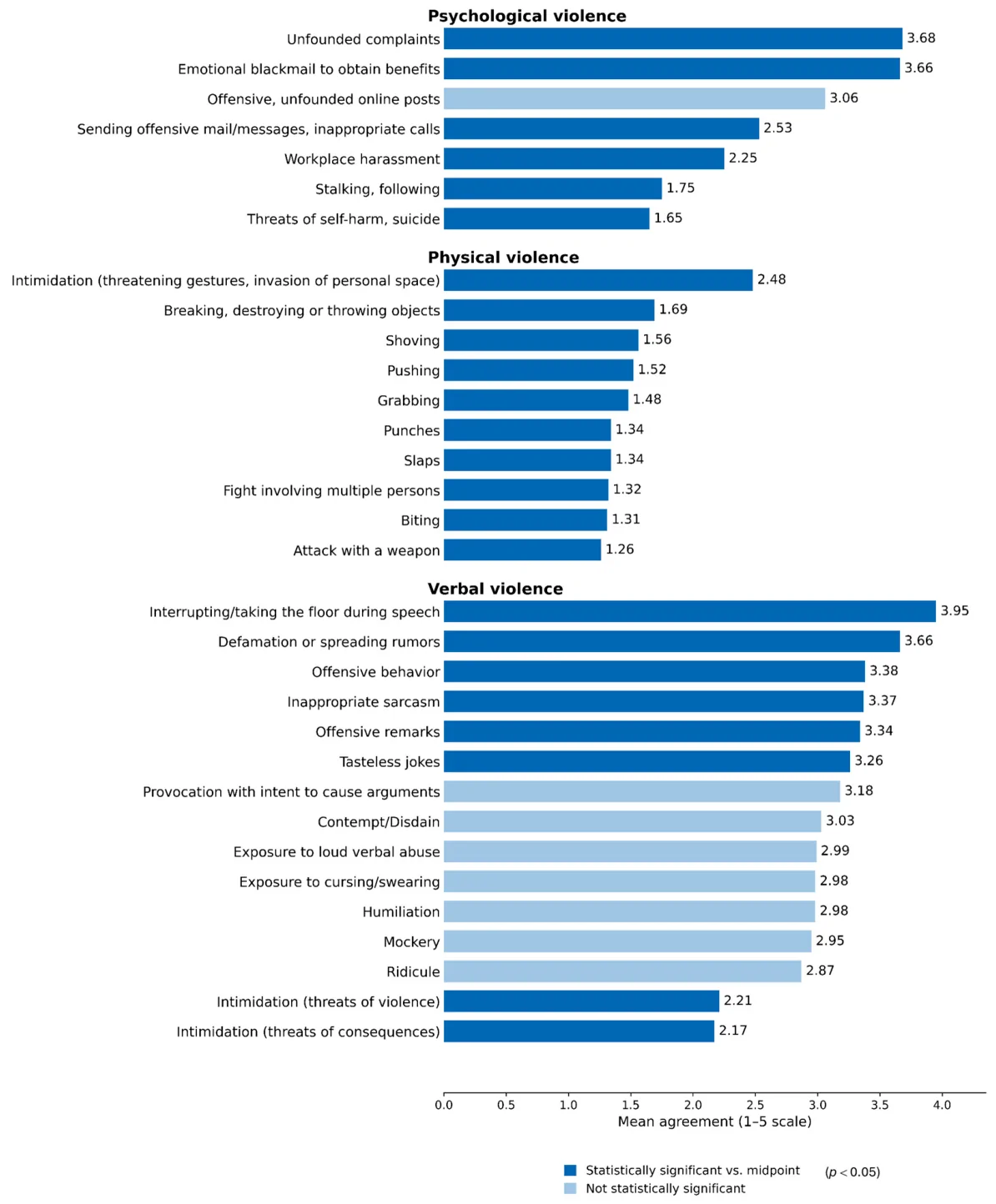
Exposure to various forms of violence by animal owners (average agreement scores are shown; respondents rated on a 5-point Likert scale; darker bars differ significantly from the midpoint 3—“neither agree nor disagree”; one-sample *t*-tests with α = 0.05).

Figure 1 shows the average agreement with experiencing violence from animal owners within veterinary institutions. The darker bars represent forms of violence for which the mean response of respondents in the one-sample *t*-test statistically significantly (α = 0.05) differs from the scale midpoint of 3 (“neither agree nor disagree”) as the middle reference value for the agreement scale. Employees in veterinary institutions report higher-than-midpoint agreement with being exposed to interruption or being talked over, slander and rumour-spreading, offensive behaviour, inappropriate sarcasm, offensive remarks, and offensive jokes from animal owners. The mean agreement scores for all these statements are statistically significantly higher than 3 (all *p* < 0.001, except for offensive jokes: *p* = 0.013). Conversely, they report less exposure to intimidation, whether in the form of threats of violence or threats of consequences, with a mean score statistically significantly lower than 3. As shown in Figure 1, employees agree less to being exposed to physical violence: the mean agreement for all listed forms was statistically significantly lower than the midpoint response of 3 (all *p* < 0.001). The greatest agreement reported among these was intimidating behaviour (threatening gestures, menacing movements, invasion of personal space), which differs somewhat from other forms of physical violence. Among the listed forms of psychological violence, respondents agreed with a mean score higher than 3 for unwarranted complaints against them and emotional blackmail to obtain benefits (crying, sobbing, etc.). They typically agreed less with sending offensive emails or messages, inappropriate phone calls, workplace harassment (frequent unwanted visits, gifts, etc.), stalking, and threats of self-harm or suicide. All *p*-values for statistically significant means were less than 0.001.

In response to the question of whether they had ever been physically injured at work by an animal owner, 95.4% of respondents reported that they had never experienced such an injury, while eight respondents reported having been physically injured. Given the small number of respondents reporting an injury (*n* = 8), these data are presented descriptively and were not subjected to further statistical analysis.

For all listed behaviours, respondents provided answers regarding both co-workers and supervisors, with mean scores statistically significantly lower than the midpoint response of 3 (*p* < 0.001). The results indicate that respondents generally report low agreement with statements indicating exposure to violence from co-workers or supervisors in the workplace. The highest agreement was observed for ignoring suggestions and opinions, withholding important information affecting work performance, and verbal violence (such as shouting). The lowest agreement was regarding exposure to sexual and physical violence in various forms. Notable differences in the distribution of forms of violence exposure between co-workers and supervisors are evident, primarily in specific behaviours that are somewhat more prominent for supervisors and rank higher on the scale than for co-workers, most notably excessive monitoring of work and demands to forgo entitlements (such as sick leave, travel expense reimbursement, a day off, etc.).

**Figure 2:**
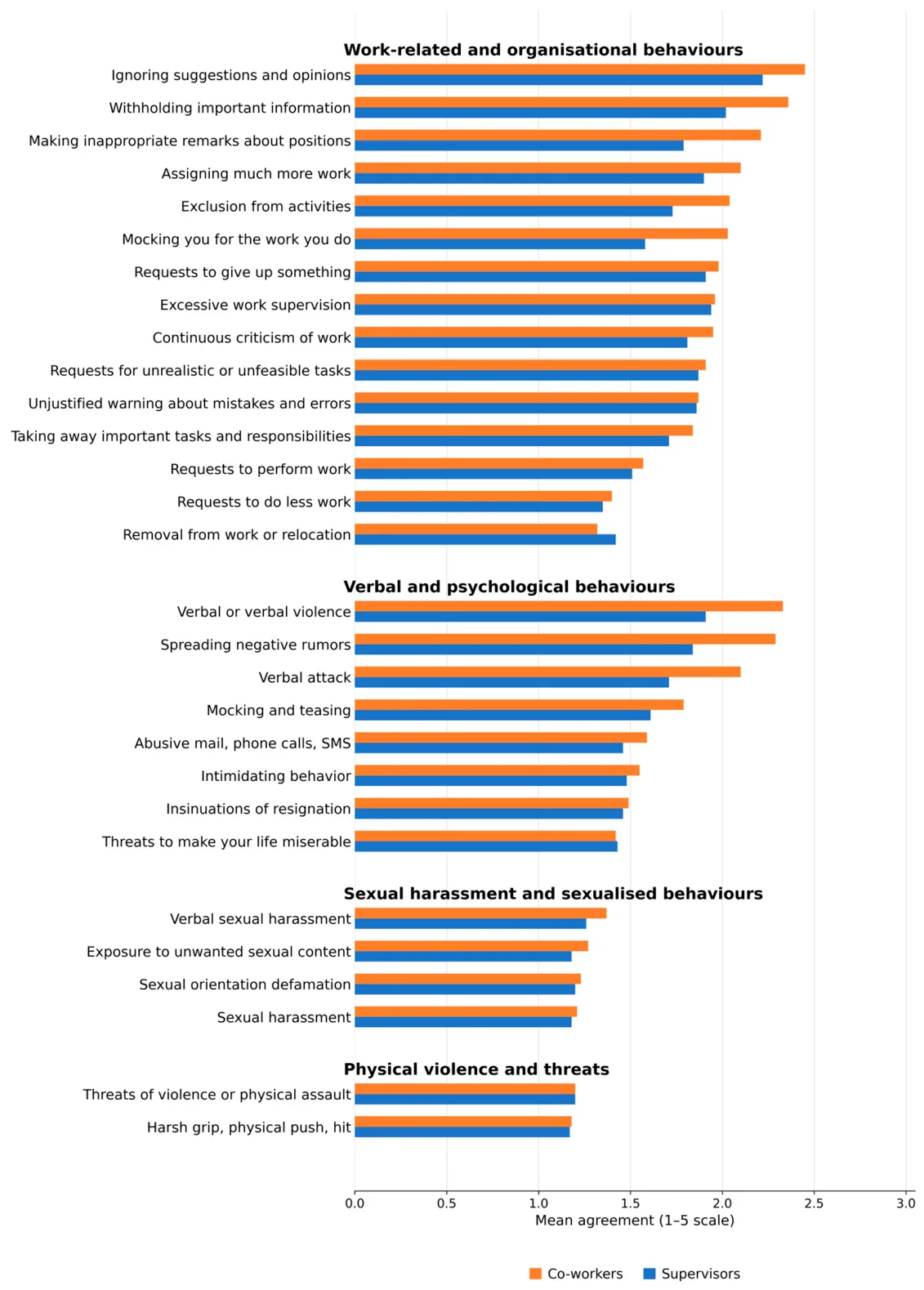
Exposure to various forms of violence from co-workers (orange) and supervisors (blue) (mean agreement scores shown; respondents rated on a five-point Likert scale).

For each perpetrator of violence (i.e., animal owners, co-workers, and supervisors) shown in Figure 2, we calculated the mean of respondents’ answers to enable a basic comparison of the agreement with experiencing general violence from different actors. The internal reliability of the resulting composite variables is very good: Cronbach’s alpha for animal owners is α = 0.957, for co-workers α = 0.960, and for supervisors α = 0.971. We therefore concluded that the composite variables obtained represent substantively coherent wholes. Still, due to somewhat different items used to identify the exposure to violence from animal owners than from co-workers and supervisors, the comparison cannot be interpreted as a direct comparison of the prevalence of the same types of violence among the perpetrator types. The comparison shown is therefore solely of an exploratory nature.

**Figure 3:**
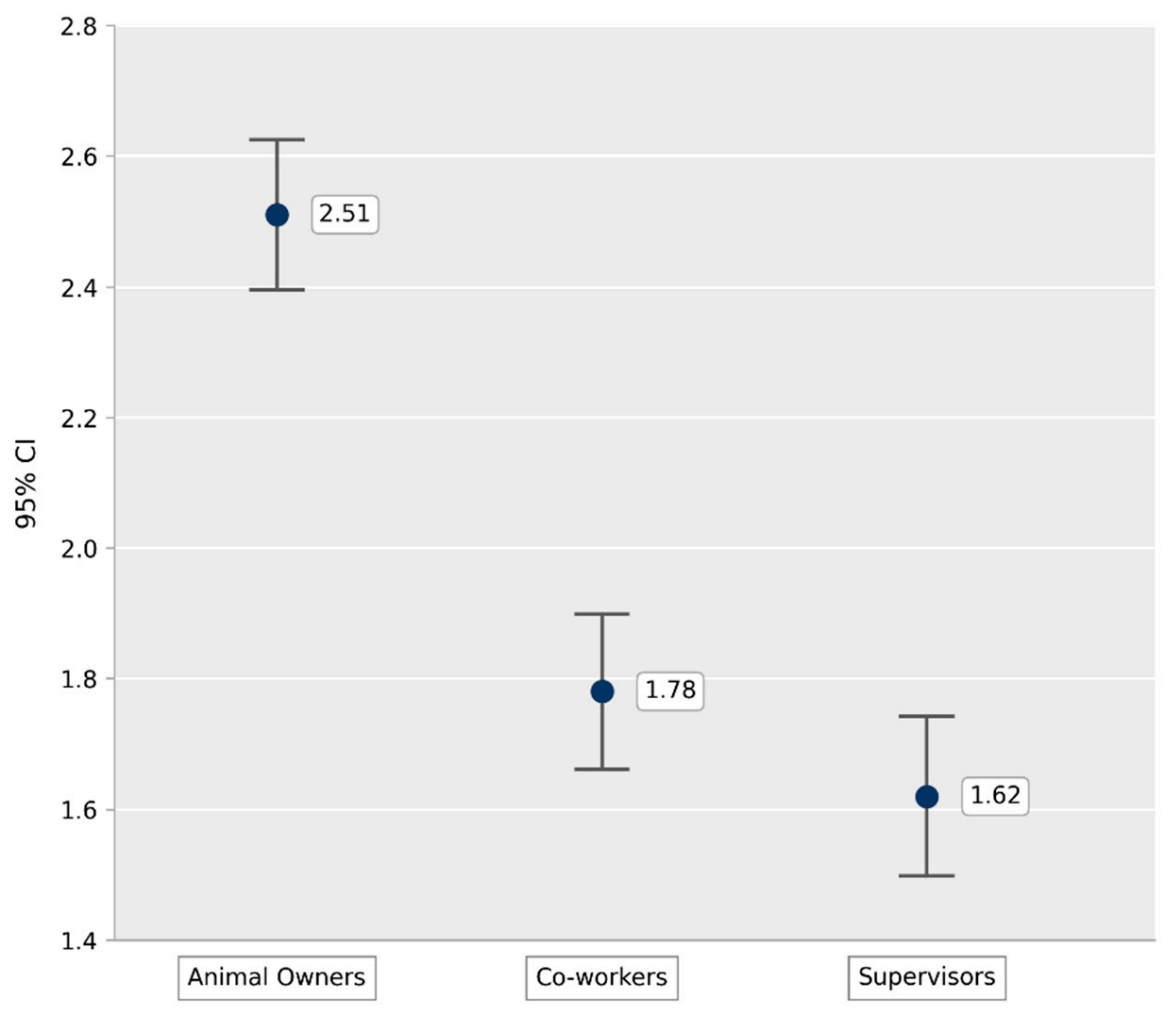
Mean agreement scores for reported exposure to violence by actor type.

We note that the variables obtained are not treated as factors, as the basic conditions for factor analysis are not met. Most indicators deviate substantially from a normal distribution. The means obtained therefore represent only composite variables for each actor, describing the mean agreement with exposure to violence in all proposed forms from these actors. All three new variables are approximately normally distributed, with kurtosis and skewness between −2 and 2. As shown in Figure 3, respondents rated the mean agreement with experiencing all forms of violence from animal owners highest (M = 2.51; SD = 0.78), considerably higher than from co-workers (M = 1.78; SD = 0.80) and supervisors (M = 1.62; SD = 0.82).

We subsequently conducted paired *t*-tests for each pair of perpetrators (Table 3) to determine whether the differences in means are statistically significant. The differences are statistically significant for all pairs: respondents, on average, agreed that they have been exposed to violence from animal owners significantly more than violence from co-workers (t = 11.04; *p* < 0.001) and from supervisors (t = 12.36; *p* < 0.001). Co-workers are also reported to be perpetrators more likely than supervisors (t = 3.98; *p* < 0.001).

### 3.2. Presence of Emotions When Confronting Violence

**Figure 4:**
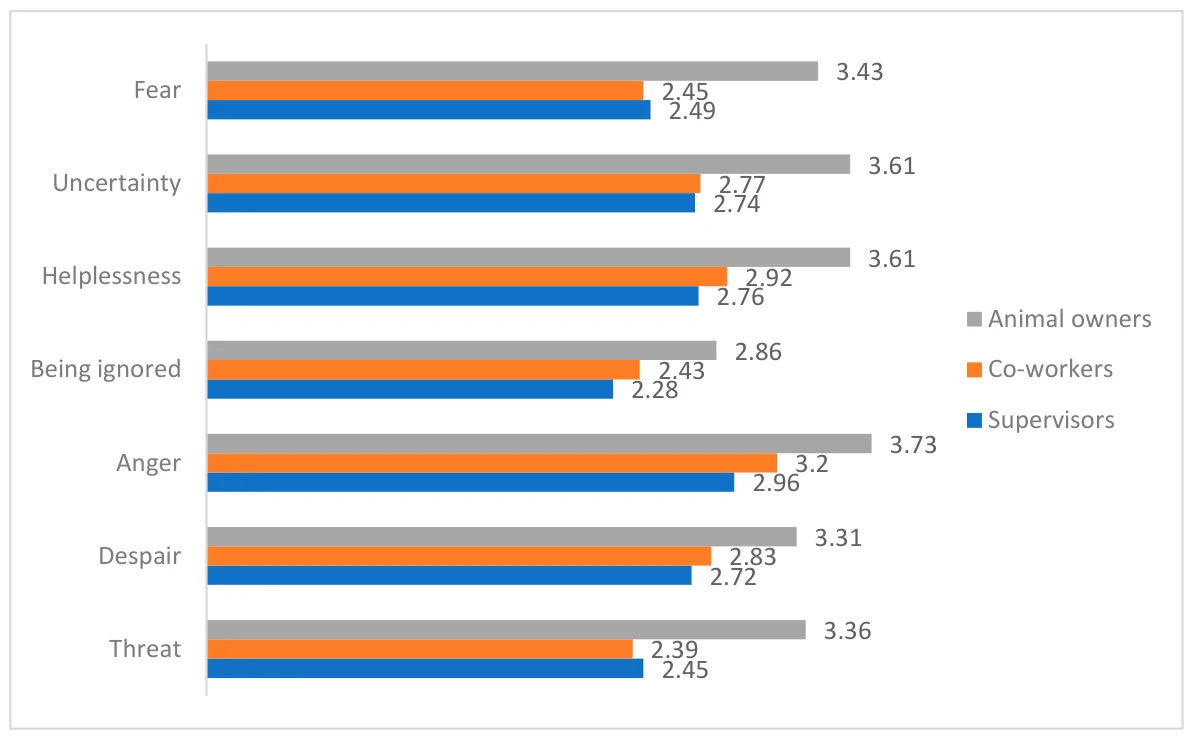
Negative emotions when experiencing violence from individual actors (mean scores of expressed emotions shown; respondents rated on a five-point Likert scale).

Figure 4 shows that respondents reported the highest mean intensity of all listed negative emotions in relation to violence involving animal owners. Mean emotional-response scores associated with co-workers and supervisors were generally lower. When comparing co-workers and supervisors descriptively, threat and fear were slightly more intense in relation to supervisors, whereas helplessness, being ignored, anger, uncertainty, and despair were somewhat more intense in relation to co-workers. Given the exploratory nature of these analyses and the large number of comparisons, these patterns are presented descriptively and should be interpreted with caution.

A multiple linear regression analysis was performed. We visually analysed the residual plots, showing no violations of linearity, homoscedasticity, and normality of residuals. VIF values did not indicate multicollinearity, with maximum values of 1.102 (animal owners model), 1.120 (co-workers), and 1.136 (supervisors). Cook’s distance calculations did not show a strong influence of observations, as they ranged from 0 to 0.070, 0.075, and 0.171 for the three models, respectively.

Education was dichotomised into lower education (secondary school or bachelor’s degree) and higher education (MSc or PhD) and entered into the regression models as a binary dummy variable, with lower education as the reference category.

The results presented in Table 4 show that at the significance level α = 0.05, the intensity of experiencing negative emotions when facing violence from animal owners (Table 4, Model 1) is statistically significantly negatively associated with work experience (B = −0.033; *p* < 0.001), higher education compared to lower education (B = −0.326; *p* = 0.023), male gender compared to female gender (B = −0.304; *p* = 0.045), and teaching staff compared to veterinarians (B = −0.797; *p* = 0.030). The group was very small (n = 7), so the findings should be interpreted with caution. We can therefore conclude that respondents with more years of work experience, those with higher education, and teaching staff experience negative emotions less intensely when confronted with violence from animal owners. Men also experience negative emotions less intensely than women.

For the experience of emotions when facing violence from co-workers (Table 4, Model 2), only work experience is statistically significantly associated at the significance level α = 0.05 (B = −0.028; *p* < 0.001), and that association is negative. Therefore, respondents with more work experience experience negative emotions less intensely when confronted with violence from co-workers.

Similarly, for the experience of negative emotions when facing violence from supervisors (Table 4, Model 3), work experience is statistically significant at the α = 0.05 level (B = −0.027; *p* = 0.006), with more years of work experience associated with less intense negative emotions. Teaching staff, in comparison to veterinarians, reported significantly higher emotional responses to violence from supervisors (B = 1.069, *p* = 0.041), although this group was very small (*n* = 7) and the findings should be interpreted with caution.

Although some coefficients for the teaching-staff subgroup reached the conventional significance threshold, this subgroup included only seven respondents; therefore, these estimates are considered unstable and are not interpreted substantively.

Respondents’ age was analysed separately and not included in the models above due to a strong correlation with work experience. Table 5 shows the unadjusted associations between emotional experiences and age to explore their relationship and should be interpreted with caution, as they can also be influenced by work experience. Results show a statistically significant negative correlation with the experience of negative emotions when facing violence from all three actors (Table 5). Older age was associated with less intense negative emotional responses to workplace violence involving animal owners, co-workers, and supervisors. However, because age was analysed separately from work experience, these associations should not be interpreted as independent age effects.

### 3.3. Perceived Knowledge, Training, and Organisational Support in Dealing with Violent Clients

Respondents agree more strongly with the statements that, for their safe work, practical workshops (M = 3.73; SD = 1.10), written action guidelines (M = 3.68; SD = 1.04), theoretical workshops (M = 3.53; SD = 1.10), and training with repeated refresher workshops (M = 3.48; SD = 1.14) are necessary.

**Figure 5:**
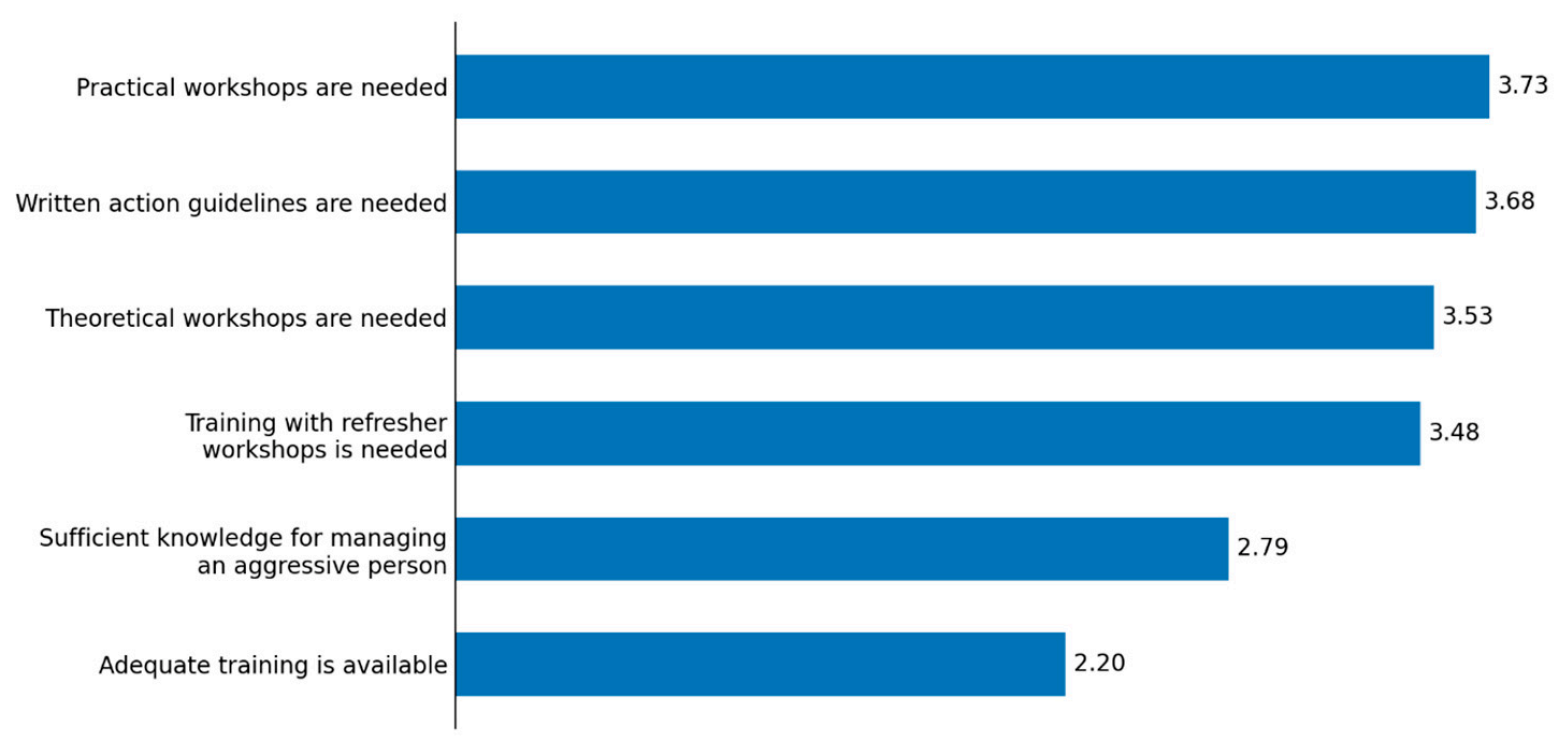
Agreement regarding perceived knowledge, training, and support in dealing with violent clients/animal owners (mean agreement scores shown; respondents rated on a 5-point Likert scale).

The one-sample *t*-test shows that the mean agreement score for all six statements differs statistically significantly from the midpoint of 3 (Figure 5). Respondents expressed relatively high agreement with the perceived need for practical workshops (M = 3.73; SD = 1.10), written action guidelines (M = 3.68; SD = 1.04), theoretical workshops (M = 3.53; SD = 1.10), and training with repeated refresher workshops (M = 3.48; SD = 1.14).

In contrast, the statements about possessing sufficient knowledge (*p* = 0.008) and the availability of adequate training (*p* < 0.001) were, on average, rated statistically significantly lower than 3.

## 4. Discussion

The results of the presented study indicate that employees of veterinary practices and clinics in Slovenia are exposed to various forms of workplace violence. Physical violence was reported less frequently, yet is not negligible, as a small proportion of respondents also reported bodily injuries. In response to the question of whether they had ever been physically injured at work by an animal owner, 95.4% of respondents answered never. This proportion appears more favourable than the 31% reported by Yukawa and Yukawa [16]. However, this comparison should be interpreted with caution, as the two studies differ in population, sampling method, item wording, and recall period, and such methodological heterogeneity limits the validity of direct cross-study comparisons of prevalence figures.

The present findings should also be considered in the context of recent veterinary research showing that inappropriate and harmful workplace behaviours may originate from both external and internal sources. Irwin et al. (2022) [10] similarly examined incivility originating from clients, co-workers, and senior colleagues in veterinary practice and demonstrated that the source of incivility may be relevant to both its perceived impact and the ways in which veterinary personnel cope with such experiences. Client incivility has also been associated with adverse occupational and emotional outcomes among veterinary professionals [9], while subsequent research has highlighted differences in responses to incivility depending on both the type of behaviour and its source [11]. More recent evidence further suggests that organisational responses and support may play an important role in how veterinary personnel experience and manage workplace incivility [12].

These findings are broadly consistent with the present study, in which reported exposure to inappropriate and violent behaviours involved animal owners, co-workers, and supervisors. However, direct comparisons between perpetrator groups in our study should be interpreted cautiously because the measures used for animal owners and those used for co-workers and supervisors were not fully equivalent. Our findings therefore support the importance of considering both the type and source of workplace mistreatment, rather than interpreting workplace violence as a single homogeneous phenomenon.

However, respondents report being exposed to verbal and psychological forms of violence, particularly from animal owners, which is consistent with findings from other studies [6,16]. For example, Yukawa and Yukawa [16] similarly identified verbal violence from animal owners as the predominant form of violence in such institutions. Similar findings are reported by Irwin et al. [12], who found that disrespect from clients was rated as the most serious and most frequently reported behaviour, while responses to co-worker behaviour were more often associated with withdrawal. We note that these studies used different measurement instruments and definitions of violence than the present survey and that our data reflect agreement with statements about exposure rather than directly measured incident frequency. The convergence across studies should therefore be read as broadly consistent rather than as confirmatory replication.

The prominent role of animal owners/clients observed in the present study is also consistent with recent veterinary research specifically examining aggression and violence originating from clients. A nationwide German survey reported substantial exposure of veterinary professionals to violence from animal owners, particularly verbal forms of aggression, demonstrating that client-related violence represents an occupational concern extending beyond the Slovenian veterinary context. Nevertheless, differences in study design, definitions, measurement instruments, and response scales limit direct comparisons of prevalence estimates between studies.

Our study also found that animal owners are frequently identified as a source of violence, while active behavioural or interventional responses were reported less often. Our study found that respondents expressed relatively high agreement with the perceived need for training, written guidelines, and organisational support. However, confidence, preparedness, and behavioural response strategies were not directly measured; therefore, the present findings do not allow conclusions about whether these factors explain responses to workplace violence. In contrast to some findings from other countries, our respondents more frequently emphasised the need for a systemic approach, including training, clear protocols, and organisational support, though again, whether such measures would in practice increase active intervention was not tested here.

A New Zealand study reported that 16% of participants experienced workplace bullying [14]. The prevalence rate in their study was similar to that in the healthcare and hospitality sectors, indicating that the veterinary profession is also not immune to negative workplace experiences in other countries. We refrain from directly comparing these figures with our results.

Workplace mistreatment in veterinary settings is not limited to interactions with clients. Recent research from the Netherlands has documented aggression, bullying, discrimination, sexual harassment, and other transgressive behaviours within veterinary workplaces, with the source of inappropriate behaviour differing according to the type of behaviour examined. These findings complement our observation that workplace mistreatment may also originate from co-workers and supervisors and underline the importance of considering internal workplace relationships alongside client-related aggression.

In the study by Fowler et al. [13], 34% of respondents reported that work-related stress affected their health and well-being, with 8% having taken sick leave as a result. More than 20% reported a lack of interest in their work, and a quarter reported feelings of depression or hopelessness. In our study, emotional responses such as fear, helplessness, and uncertainty were most frequently reported following violence from clients. We did not directly assess depression, anxiety, burnout, sick leave, or other indicators of psychological morbidity. Therefore, our findings should not be interpreted as evidence of deteriorating mental health. Rather, they indicate that specific negative emotions were reported in association with specific exposure to violence. Similarly, greater work experience and, in cases of violence by animal owners, higher education and male gender were associated with less intense negative emotional responses. We interpret this cautiously: although one possible explanation is that experience and education are linked to greater professional confidence, confidence was not measured in this study. The observed associations could equally reflect other unmeasured factors, such as seniority, differences in client-related responsibilities, or the selective removal of less experienced staff from roles involving greater client interaction. We therefore present this as one of several plausible explanations rather than an established mechanism.

It is important to emphasise that violence occurs not only in interactions with service users but also within the organisation, as shown by reports of violent behaviour from co-workers and supervisors. Irwin et al. [12], for example, found that employees are more likely to report deviant behaviour from a client, animal owner, or co-worker than from a supervisor. This may reflect differential willingness to report rather than differential exposure.

Violence and threatening situations affect not only employee safety but also place a significant burden on their mental health and emotional stability. Phenomena such as sleep disturbances, feelings of loneliness, and overload are directly associated with exposure to threats and physical attacks, as well as a sense of helplessness, especially when employees work alone [7,13]. We did not measure these outcomes directly, but the emotional responses observed in our sample are broadly consistent with this literature.

Nevertheless, respondents reported somewhat low mean agreement with the statement that they possess enough knowledge to manage violent situations. There was strong support for the introduction of written guidelines and both practical and theoretical workshops, ideally delivered as regular training. Some other studies have similarly emphasised the value of structured education and training for veterinary staff and students [25], including a half-day training programme and strengthening cooperation between veterinary clinics and other stakeholders involved in Sweden. The importance of ongoing education and training for effective responses to identified violence is also emphasised by Yukawa and Yukawa [16]. Along with clear action guidelines and assigned responsibility during interventions, these measures can positively affect the reduction in burnout and stress among veterinary employees [26]. Additionally, it is important to note that our data indicate only that respondents expressed a preference for these measures. Our study did not evaluate whether training, protocols, or organisational and psychological support reduce exposure to violence or its emotional impact, and such conclusions should not be drawn solely based on the respondents’ stated references. Evaluating such interventions would require dedicated research designs, ideally longitudinal or intervention-based.

## 5. Conclusions

The results of this study provide important insight into the psychosocial risks of workplace violence faced by employees of veterinary practices and clinics in Slovenia. Given the lack of existing research in this area within the Slovenian context, these findings contribute to a better understanding of working conditions and challenges related to workplace violence in the veterinary profession.

The findings indicate the need for measures at multiple levels. The findings suggest a potential need for measures at multiple levels. Based on the results, regular training and education on recognising and handling violent situations could be considered a potentially beneficial measure for staff in veterinary practices and clinics. In addition, the findings of the study suggest that access to professional psychological support could be considered a potential measure to support employees exposed to violence. However, since this study did not evaluate any interventions, it cannot determine whether such measures would, in practice, reduce exposure to workplace violence or mitigate its psychological consequences. Therefore, we treat them as needs identified by the participants and as potential avenues for future intervention research, rather than as evidence-based recommendations for practice.

Despite the important findings of the study, some methodological limitations must be acknowledged, such as the self-report nature of the questionnaire, which relies on the subjective perceptions and experiences of individuals. This approach is common in studies of phenomena such as workplace violence or other forms of deviant behaviour, but there is a possibility that respondents did not report certain experiences; for example, they may have forgotten or suppressed them. Additionally, the instrument measured agreement with exposure to violence, not its frequency. Due to the not fully equivalent measurement structure across perpetrator groups, differences in mean scores may partly reflect this issue and do not represent true differences in exposure to violence from those three groups of perpetrators. Also, the large number of statistical tests increases the possibility of Type I error. Because no formal multiplicity adjustment was conducted, individual *p*-values should be interpreted with caution. Another limitation concerns the understanding of the term ‘workplace violence’, as this is interpreted differently in various societies and countries. An additional limitation is the possibility of sampling bias, particularly non-response and self-selection bias due to the rather low response rate (13.17%), as participation in the study was voluntary, meaning that individuals with personal experience of violence or who considered the topic particularly important may have been more motivated to participate. The sample size or power calculation was not previously conducted. The representativeness of the sample could not be established due to the low response rate, possible sampling bias, and the absence of detailed demographic data for all of the Slovenian veterinary employees, so the results have limited generalisability. Furthermore, some sociodemographic and professional subgroups within the sample were very small, which limits the statistical power and reliability of associations involving these subgroups; therefore, the corresponding results should be interpreted with particular caution. Certain discrepancies were also observed in the number of missing responses for individual items and variables; this may have affected the comparability of results across different analyses and should be taken into account when interpreting the findings. The cross-sectional design also precludes conclusions about temporal or causal relationships between reported workplace violence, respondent characteristics, and emotional responses. Furthermore, because both exposure and emotional responses were assessed using the same self-report questionnaire, the observed associations may be affected by common-method bias.

Nevertheless, despite these limitations, the study offers relevant insight into a specific and previously neglected research area and opens possibilities for further research, as it contributes to greater awareness and directs the attention of the profession and relevant institutions towards the development of safer and more supportive working conditions in veterinary practice.

Future research should address the methodological limitations identified in the present study by using validated, event-based measures of workplace violence with clearly defined recall periods and by including larger and more representative samples. Incorporating organisational-level factors may provide further insight into workplace characteristics associated with different forms of violence. Longitudinal studies are needed to examine the consequences of workplace violence over time, while intervention studies should evaluate whether measures such as training, written protocols, and organisational or psychological support are effective in reducing workplace violence and its adverse consequences.

## Figures and Tables

**Table 1:** Sample description.

		f (%); M (SD)
Gender		
	Female	123 (69.5)
	Male	54 (30.5)
Mean age (SD) *		41.9 (11.7)
Education		
	Secondary school	9 (5.1)
	Bachelor’s degree	106 (59.9)
	MSc	42 (23.7)
	PhD	20 (11.3)
Position		
	Veterinarian	155 (87.1)
	Professional associate	16 (9.0)
	Teaching staff	7 (3.9)
Average years of work experience (SD) *		16 (11.4)

* Both numerical demographic variables are approximately normally distributed.

**Table 2:** Components and loadings after oblique rotation >0.40.

		Comp. 1	Comp. 2	Comp. 3
Owners				
	Fear		0.780	
	Uncertainty		0.749	
	Helplessness		0.854	
	Being ignored		0.552	
	Anger		0.559	
	Despair		0.798	
	Threat		0.751	
Co-workers				
	Fear			0.618
	Uncertainty			0.886
	Helplessness			0.819
	Being ignored			0.832
	Anger			0.560
	Despair			0.694
	Threat			0.565
Supervisors:				
	Fear	0.815		
	Uncertainty	0.811		
	Helplessness	0.887		
	Being ignored	0.506		
	Anger	0.783		
	Despair	0.846		
	Threat	0.903		

**Table 3:** Results of paired *t*-tests for perpetrators.

Pairs	N	M	SD	t	*p*	Cohen’s d	95% CI Lower	95% CI Upper
Animal owners	177	2.51	0.78	11.044	<0.001	0.830	0.658	1.000
Co-workers		1.77	0.80					
Animal owners	173	2.51	0.78	12.357	<0.001	0.940	0.760	1.118
Supervisors		1.62	0.82					
Co-workers	174	1.78	0.81	3.979	<0.001	0.302	0.149	0.453
Supervisors		1.63	0.82					

**Table 4:** Multiple linear regression models of emotional experience.

Dependent Variable	Independent Variables	B	β	t	*p*	95% CI Lower Bound	95% CI Upper Bound
Model 1: Emotions—animal owners R^2^ = 0.266	Constant	4.187		33.596	<0.001	3.941	4.433
Adjusted R^2^ = 0.244	Work experience	−0.033	−0.378	−5.384	<0.001	−0.045	−0.021
N = 170	Education = Higher	−0.326	−0.158	−2.289	0.023	−0.607	−0.045
F = 11.888	Gender = Male	−0.304	−0.142	−2.016	0.045	−0.602	−0.006
*p* < 0.001	Group = Professional associate	0.1	0.028	0.414	0.679	−0.376	0.576
	Group = Teaching staff	−0.797	−0.151	−2.190	0.030	−1.516	−0.078
Model 2: Emotions—co-workers R^2^ = 0.103	Constant	3.156		18.997	<0.001	2.827	3.484
Adjusted R^2^ = 0.075	Work experience	−0.028	−0.270	−3.425	<0.001	−0.044	−0.012
N = 167	Education = Higher	0.023	0.009	0.117	0.907	−0.359	0.405
F = 3.702	Gender = Male	−0.122	−0.047	−0.590	0.556	−0.530	0.286
*p* = 0.003	Group = Professional associate	0.347	0.081	1.073	0.285	−0.292	0.987
	Group = Teaching staff	0.860	0.145	1.887	0.061	−0.04	1.759
Model 3: Emotions—supervisors R^2^ = 0.094	Constant	3.135		16.756	<0.001	2.766	3.505
Adjusted R^2^ = 0.066	Work experience	−0.027	−0.226	−2.807	0.006	−0.046	−0.008
N = 166	Education = Higher	−0.038	−0.013	−0.171	0.865	−0.472	0.397
F = 3.339	Gender = Male	−0.264	−0.091	−1.13	0.260	−0.726	0.197
*p* = 0.007	Group = Professional associate	−0.002	0.000	−0.006	0.995	−0.704	0.699
	Group = Teaching staff	1.069	0.161	2.064	0.041	0.046	2.092

**Table 5:** Results of the correlation between age and emotional experience.

		Animal Owners	Co-Workers	Supervisors
Age	Pearson correlation	−0.398	−0.256	−0.237
	*p*	<0.001	<0.001	0.002
	n	169	165	164

## Data Availability

The data presented in this study are available on request from the corresponding author. The data are not publicly available to protect the privacy and confidentiality of participants, given the potentially sensitive nature of the survey data.
